# Joint Driver State Classification Approach: Face Classification Model Development and Facial Feature Analysis Improvement

**DOI:** 10.3390/s25051472

**Published:** 2025-02-27

**Authors:** Farkhod Akhmedov, Halimjon Khujamatov, Mirjamol Abdullaev, Heung-Seok Jeon

**Affiliations:** 1 Department of Computer Engineering, Gachon University, Seongnam 13120, Gyeonggi-Do, Republic of Korea; farhod34@gachon.ac.kr (F.A.); khujamatov@gachon.ac.kr (H.K.); 2 Department of Information Systems and Technologies, Tashkent State University of Economics, Tashkent 100066, Uzbekistan; abdullaevm@tsue.uz; 3 Department of Computer Engineering, Konkuk University, 268 Chungwon-daero, Chungju-si 27478, Chungcheongbuk-do, Republic of Korea

**Keywords:** image processing, image restoration, face analysis, landmark application, drowsiness detection

## Abstract

Driver drowsiness remains a critical factor in road safety, necessitating the development of robust detection methodologies. This study presents a dual-framework approach that integrates a convolutional neural network (CNN) and a facial landmark analysis model to enhance drowsiness detection. The CNN model classifies driver states into “Awake” and “Drowsy”, achieving a classification accuracy of 92.5%. In parallel, a deep learning-based facial landmark analysis model analyzes a driver’s physiological state by extracting and analyzing facial features. The model’s accuracy was significantly enhanced through advanced image preprocessing techniques, including image normalization, illumination correction, and face hallucination, reaching a 97.33% classification accuracy. The proposed dual-model architecture leverages imagery analysis to detect key drowsiness indicators, such as eye closure dynamics, yawning patterns, and head movement trajectories. By integrating CNN-based classification with precise facial landmark analysis, this study not only improves detection robustness but also ensures greater resilience under challenging conditions, such as low-light environments. The findings underscore the efficacy of multi-model approaches in drowsiness detection and their potential for real-world implementation to enhance road safety and mitigate drowsiness-related vehicular accidents.

## 1. Introduction

Detecting driver drowsiness stands as a critical issue for upholding road safety [1], addressing instances where drivers navigate vehicles while grappling with fatigue, somnolence, or diminished alertness levels due to the driver’s physical state. Such drowsiness can emanate from a multitude of factors, ranging from encompassing insufficient rest, prolonged periods of driving, and unstimulating road environments. Recent research achievements reveal that the accuracy of driver face detection systems is significantly affected mainly by environmental conditions, driver movements, obstructions within the vehicle, as well as external factors, like the quality of camera recording. All these factors collectively present challenges for face detection algorithms, influencing their ability to reliably identify and monitor drivers in real-world scenarios. Overcoming these challenges requires advancements in technology, such as improved camera systems and algorithms capable of handling diverse conditions and driver behaviors, to enhance the effectiveness of driver monitoring systems and to avoid driver-related accidents.

Cutting-edge DL methodologies and computer vision (CV) algorithms have emerged as a potent strategy for discerning the well-being of drivers. Leveraging DL to decipher intricate patterns within visual data facilitates the development of resilient and precise detection models. However, in the realm of vehicular operation, facial recognition systems encounter formidable challenges rooted in the diverse array of environmental variables. These challenges encompass, but are not limited, fluctuations in illumination conditions, spanning from stark shadows to glaring sunlight and sudden shifts in ambient lighting [2]. The dynamic nature of these environmental elements poses formidable obstacles to the consistent and accurate identification of drivers’ facial attributes amid real-world driving scenarios. Moreover, recording a driver’s face and extracting the main facial features to analyze a driver’s face are overwhelming during nighttime driving as well. Models find it difficult to classify a driver’s state or may classify it wrongly. Addressing these challenges mandates a holistic consideration of adaptive methodologies within facial recognition algorithms aimed at ameliorating the deleterious impact of environmental variability on the dependability and efficacy of facial recognition systems in on-road contexts. Furthermore, within the domain of CV, the realm of image restoration assumes paramount importance for a myriad of reasons, serving as a linchpin in augmenting the quality and reliability of visual data.

In face detection, facial feature analysis constitutes a critical aspect, involving the identification of structural attributes that remain consistent across variations in poses, viewpoints, and lighting conditions. Recently, researchers introduced a novel image enhancement algorithm [3] aimed at enhancing face detection performance in non-uniform lighting conditions and in high-dynamic-range environments. Drawing inspiration from established principles such as multi-scale retinex with color restoration and the illumination reflectance model-based nonlinear enhancement, this approach prioritizes efficient dynamic range compression to elevate face detection efficacy. Additionally, innovative frameworks for event-based driver face detection have emerged, leveraging event data processing patterns to facilitate efficient and precise feature extraction [4]. These frameworks employ translation-invariant backbones and integrate shift FPN and shift context modules to enable the exchange of temporal neighbor information among events. So, efforts have been directed towards detecting a driver’s face in low-light conditions, particularly with infrared lighting systems [5,6]. Techniques like adaptive attenuation quantification retinex (AAQR) have been proposed to enhance image details captured during nighttime, providing tailored solutions for driver face detection applications [5]. By integrating landmark estimation and affine transformations, these approaches enhance nighttime face images while enabling the real-time monitoring of driver states and the delivery of relevant services within practical application systems. However, several challenges must be addressed to effectively detect and analyze drivers’ faces, particularly low-light control. Low-light images refer to photographs or digital images captured under poorly illuminated conditions.

Low-light image control and enhancement is a burgeoning field within single-image enhancement techniques that has gained considerable interest among researchers [2,7], prompting an intensified focus on its exploration. In our research, a driver’s face is mainly illuminated at nighttime by vehicle headlights. In such scenarios, the available light may be insufficient for the camera sensor to effectively capture facial details, such as mouth and eye landmarks. This can potentially result in facial captures with reduced brightness, increased noise, and limited visibility. In other words, low-light images typically exhibit lower contrast, poorer color rendition, and higher levels of visual noise compared to images captured under well-lit conditions. These conditions commonly occur in indoor environments, such as inside vehicles at night or in areas with limited ambient light, presenting challenges for conventional imaging systems to generate high-quality images with sufficient clarity and fidelity. Therefore, we considered to focus on low-light image control for drivers’ faces in both daytime and nighttime. The initial approaches to low-light image enhancement primarily focused on uniformly amplifying the illumination across the entire image. Subsequent advancements involved techniques that aimed to adjust global illumination properties and learning-based methods [8,9,10] in diverse conditions. In addition to the low-light control step, we also intend to focus on the image normalization process. The literature [11] suggests that the fluctuations in signal intensities observed within the same facial images due to differing illumination conditions frequently surpass the alterations in facial structural characteristics that define identity. Illumination normalization techniques help mitigate variations in lighting conditions that may affect the visibility of facial features in driver images. By equalizing or adjusting the illumination across images, we can enhance the visibility of facial landmarks, such as eyes, nose, and mouth, making them more distinguishable for detection algorithms. Landmark localization involves identifying specific points or regions on a driver’s face, such as the corners of the eyes, the tip of the nose, or the edges of the mouth.

Overall, driver drowsiness is a critical issue in road safety, contributing to a significant number of accidents worldwide. Identifying drowsiness in drivers is essential for preventing accidents and ensuring road safety. Traditional methods for detecting driver drowsiness have relied on physiological signals such as eye closure duration and head movements, and these techniques remain highly applicable as they perform well in handling low-light, low-resolution images. However, these traditional methods cannot outperform, in terms of image restoration, recently developed machine learning algorithms. Therefore, in this paper, we present a novel approach for driver drowsiness detection model performance improvement by combining traditional methods with state-of-the-art methods, and our contributions are as follows:

1. Face image normalization in scenarios affected by destructive light;
2. Retain a driver’s face identity using a generative facial image prior in image restoration;
3. Advance drowsiness classification model accuracy through facial landmark analysis.

**Figure 1:**
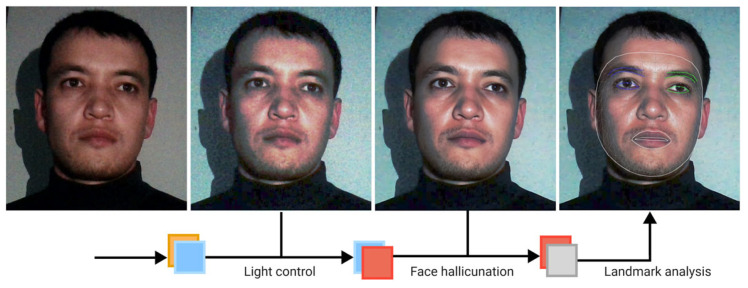
Illustration of proposed processes from an input of a low-light face image to the facial landmark application stage.

Figure 1 below is representative framework of whole process that we go through in this study, starting from low resolution, and low light nighttime input image to normalization, restoration and landmark application steps for proper analysis of driver’s state.

## 2. Related Works

**Figure 2:**
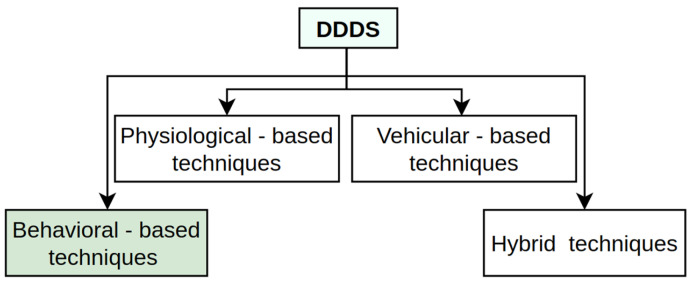
Overview of the four most common DDDS techniques.

In the context of the driver drowsiness detection system (DDDS) [12], several techniques have emerged that fall under distinct categories, as shown in Figure 2, each with its unique strengths and limitations. A physiological-based technique monitors biological signals like heart rate, eye movement, brain activity, and skin conductance to assess driver fatigue [13,14].

This approach is highly accurate as it directly measures the physiological changes associated with drowsiness, making it less prone to eternal environmental factors like lighting or obstructions. However, it often requires intrusive or wearable sensors, which can make it uncomfortable for drivers and may limit their practical implementation. Additionally, the cost of hardware and the complexity of signal processing are higher compared to other methods like visual or behavioral monitoring systems. According to vehicular-based driver drowsiness detection techniques, several research works [14,15,16,17] that analyze the implementation of vehicle monitoring systems inform us about irregularities, such as braking behavior, lane deviation, wheel movements, and others. It should be mentioned that both techniques have their advantages, and they can be seen in hybrid applications, where the DDDS combines all available methods. A behavioral-based approach is also one of the widely studied research field by other researchers [12,18], which mostly focuses on vision-based technique applications. While drivers are on the road, their faces should be analyzed all day. In this study, our focus is to deal with input images where a driver’s face is dark and illuminated with low light at low resolution because of unstable recording and other factors.

Back-lit image controlling methods are a type of image processing technique designed to address the challenges posed by back-lit or strongly illuminated scenes. In back-lit scenarios, the primary light source is located behind the subject, causing the subject to appear dark or underexposed in the captured image due to the camera’s exposure settings being optimized for the bright background. These algorithms aim to adjust the exposure and brightness levels of the captured image to ensure that both the subject and the background are properly exposed and visible. They typically involve analyzing the image to identify regions that are underexposed due to back lighting and then applying appropriate adjustments, such as contrast compensation by fuzzy classification [19] to enhance the visibility of these regions while preserving the overall image quality.

Some common techniques used in back-lit image controlling algorithms include histogram equalization (HE). This technique redistributes the intensity values of pixels in the image histogram to achieve a more balanced distribution of brightness levels. It can help enhance the visibility of dark regions in back-lit images without overexposing the bright areas. Back-lit image controlling algorithms are widely used in various applications, including photography, surveillance, computer vision, and automotive safety systems. They help improve the visibility and clarity of subjects in challenging lighting conditions, enabling better analysis, interpretation, and understanding of visual information captured in back-lit environments. Additionally, these algorithms contribute to the enhancement of the overall quality and usability of images captured in adverse lighting conditions, thereby facilitating more accurate decision-making and analysis in various domains.

### 2.1. Illumination Normalization

Illumination normalization (IN) in face detection refers to the process of correcting variations in illumination across facial images to improve the accuracy of face detection algorithms. Illumination variations can result in inconsistent brightness and contrast levels, making it challenging for face detection systems to identify facial features accurately. The main goal of illumination normalization is to enhance the visibility of facial features by reducing the impact of uneven lighting conditions. Illumination normalization techniques are essential for addressing illumination issues while preserving the original features of an image. Among these methods, illumination normalization approaches are highly valued for their ability to correct illumination problems without compromising the inherent characteristics of the image [20]. Some of the most commonly employed techniques renowned for their simplicity include HE and gamma intensity correction (GC) [21]. Image normalization refers to the process of standardizing the pixel values of an image to a common scale or range. This technique is commonly used in image processing and computer vision tasks to ensure consistency and improve the performance of machine learning models. Thus, image normalization is a crucial step in our research, referring to the standardization of pixel values to achieve a common scale and distribution. The purpose of image normalization is to remove variations in brightness, contrast, and color balance between frame iterations, making them more consistent and comparable for subsequent analysis. The techniques we are using for image normalization include mean subtraction, standard deviation normalization, and min-max scaling. Finally, and importantly, for facial features, we apply landmarks. Illumination normalization can aid in accurately localizing these landmarks by reducing the impact of shadows, highlights, and uneven illumination, which might otherwise obscure or distort the appearance of facial features. Driver monitoring systems often operate in diverse environmental conditions, which include varying lighting levels, glare, and shadows. Illumination normalization techniques can help standardize the appearance of facial features across different lighting conditions, improving the robustness of facial feature detection and landmark localization algorithms. Proper application is the key to achieve a higher accuracy of detection and classification. Therefore, we aimed to include illumination normalization for driver face image analysis. Researchers [22] have used IN techniques to detect faces by focusing on an edge-based approach for non-uniform illumination conditions. The proposed method includes red, green, and blue (RGB) color channel normalization [23], GC to control for the brightness of the input image, and fusion for both of the above techniques. Many extended studies [24,25,26] have been proposed to tackle face detection issues under extreme lighting conditions. Chen et al. [27] used an image decomposition methodology to partition an image into distinct layers, delineating a large-scale layer capturing illumination-dependent information and a detail-scale layer representing illumination-independent aspects. This approach holds particular significance within the realm of face recognition research, where mitigating illumination variations is imperative for obtaining reliable and illumination-invariant features.

Additionally, alternative methodologies have been explored to extract illumination-insensitive features by employing a sequence of image processing steps including gamma correction, difference of Gaussians, masking, and contrast equalization [28]. Furthermore, a novel normalization strategy was proposed, wherein the normalization process was applied separately to large- and small-scale features extracted from the original input face image [29]. This multi-step normalization approach aimed to bolster the robustness of the recognition system by alleviating the influence of illumination variations. However, it is noteworthy that these advanced techniques entail intricate processing steps, each of which may introduce complexities that could impact the final recognition outcomes. The proposed generative adversarial network (GAN) [29] is a powerful framework that has been widely applied for generating sharp and realistic images with many GAN-related networks [30,31,32]. This is because GANs enable the generator to acquire knowledge of a distribution that closely resembles the distribution of real data. Ling et al. [33] proposed a multi-stage feature map (MSFM) for face image IN. The researchers’ method includes generative (*G*) and discriminative (*D*) structures, where *D* distinguishes the image *G*(*x*) and corresponds with the ground truth *y*. Here, x is a poor-lighted face and y is the face with standard illumination. What they expected is that the generated image *G*(*x*) is identical to its corresponding ground truth y in the case of similar illumination and identity application. Han et al. [34] developed an asymmetric joint GAN architecture to normalize face images under arbitrary illumination conditions without knowing the face geometry and albedo information. Histogram equalization is one fundamental image processing technique used to enhance the contrast of an image by adjusting the intensity distribution across its histogram. In the context of facial feature detection, histogram equalization plays a crucial role in improving the visibility and clarity of facial features, which are essential for accurate detection and analysis [35]. By redistributing pixel intensities across the entire dynamic range, HE enhances the contrast of facial images. This results in better differentiation between facial features and the background, making it easier for detection algorithms to identify key landmarks such as eyes, nose, and mouth [36]. Moreover, HE is a versatile technique that can be helpful to equalize uneven lighting and illumination variations (Algorithm 1).

The HE is gray on the image x, and the value *i* is a gray level equal to a maximum value of 255, whereas to look for the probabilities of a pixel image from level I in the image, the following is calculated:

(1) $$P_{x}\left(i\right)=P_{\left(x=1\right)}\frac{n_{j}}{n},\;\;0\leq i<L$$

**Algorithm 1.** HE FunctionRequire: image input is the original image, and the image input is converted to a grayscale image.Ensure: image output is the image histogram equalized from the image input via the following steps:1: Use the function to read the image;2: Turn the result into an equalized variable with convert image to equalize the histogram;3: Stack the images side-by-side;4: Show the resulting image.

The variable *L* is the number of gray levels present at the image 0≤i<L.

The variable n is the number of pixels from the dataset of the image.

The variable $P_{x}\left(i\right)$ is a representation of the image histogram for the pixel into an index to *i*.

Ping et al. [37] proposed oriented local HE (OLHE) for face recognition. OLHE’s application was similar to LHE using the difference in orientation to edges. Reference [38] proposed the contrast-limited adaptive HE (CLAHE) method for face recognition in low-light conditions, and the study achieved remarkable results for face recognition. The authors performed the HE on the local ω-by-h window centering on the pixel as follows:

(2) $$f\left(x\right)=round\left[\frac{cdf\left(x\right)-{cdf}_{min}}{\left(\omega \cdot h\right)-{cdf}_{min}}\;\cdot (L-1)\right]$$

where *x* represents the pixel intensity value, $cdf\left(x\right)$ is the cumulative distribution function of the pixel intensities in the ω-by-h window.${\;cdf}_{min}$ is the minimum intensity in this window and L is the output gray levels.

Refs. [39,40,41,42] proposed and evaluated the CLAHE technique for face recognition, and their studies underscore the pivotal role of HE application in enhancing face recognition efficacy.

### 2.2. Face Hallucination

The challenges associated with high-resolution images are strongly correlated with low-resolution images, particularly in the context of facial analysis. These challenges have significant implications for driver monitoring systems aiming to restore facial details and detect the driver’s physical state. Low-resolution images pose multifaceted obstacles in facial analysis, notably affecting face detection, recognition, and feature extraction tasks. The loss of fine details inherent in low-resolution images impedes the accurate identification of crucial facial landmarks such as eyes, nose, and mouth. This compromises the efficacy of facial recognition algorithms reliant on discriminative features, leading to increased false positives and negatives in driver monitoring applications.

Moreover, the challenges extend to localization tasks, where the lack of clear boundaries and landmarks complicates the precise detection and alignment of facial features, which are critical for assessing the driver’s physical state. In the realm of biometric applications, low-resolution facial images fail to meet stringent accuracy requirements, introducing vulnerabilities in identity verification systems that are crucial for driver authentication and access control. Addressing these challenges necessitates advanced image restoration techniques tailored for driver monitoring systems. Leveraging advancements in image super-resolution and enhancement methodologies can help restore facial details, mitigate the impact of low-resolution images, and enable more accurate detection of the driver’s physical state. By enhancing image quality and extracting crucial facial features, these techniques facilitate more robust and reliable driver monitoring systems, which is crucial for ensuring road safety.

Face hallucination (FH) is a representation of the super-resolution (SR) problem, where the main goal is to obtain high-quality facial images from low-quality inputs [43]. It is widely recognized that the FH technique can generate imagery or information from a given input image across various modalities, including different resolutions, styles, or imaging modes. The application of SR has a wide range of applications across various fields, such as medical imaging [44,45,46,47], surveillance, and security.

These models leverage advanced ML and computer vision algorithms to enhance and transform facial images, and image enhancement can improve the detection and recognition of facial features even in challenging conditions. For example, in scenarios where camera vibrations or other factors degrade the quality of input images, FH techniques can compensate for the loss of detail by generating high-resolution versions of the images, which can facilitate more precise localization of facial features. There are numerous FH technique applications that have been applied to face detection, face recognition, face editing, and 3D face reconstruction [48,49,50,51], where researchers upscale low-resolution images. Ref. [43] mentioned that previous works were mainly categorized into three main streams, namely holistic-based, part-based, and DL-based methods. Wang et al. [52] proposed a linear mapping super-resolution approach between low-resolution (LR) and high-resolution (HR) images to achieve better intensity on the face. The proposed method used an eigen transformation of LR faces, relying on a statistical model derived from a large dataset of facial images, such as an eigen transformation employed to LR faces to capture essential facial features, and a linear mapping function was established between LR and HR images to enable the model to predicate HR details by comparing the LR levels. Another linear mapping method was proposed by Liu et al. [53], which involved the integration of bilateral filtering. This method aims to reduce ghosting artifacts and helps to preserve edges and fine details while smoothing out noise and artifacts.

Recently, DL-based face SR methods have been actively explored. These implications leverage sophisticated neural network architectures to enhance the resolution of LR face images, producing highly detailed and realistic HR results. For example, Cao et al. [54] designed an attention-aware mechanism and a local enhancement network to alternately improve image regions in SR by focusing on critical facial features, such as eyes, nose, mouth, in the application of targeted area enhancement. Ye et al. [50] developed a GAN-based model to hallucinate very LR face images. The proposed approach applied GANs to generate HR facial images from LR input images by learning complex mappings between the input and output domains. In this process, adversarial training frameworks were helpful in producing images that are perceptually convincing and rich in detail. However, as mentioned by [46], these methods focus on super-resolving near-frontal LR faces, which leads to restrictions when handling small pose variabilities.

Despite significant advancements in face SR techniques, previous methods exhibit several limitations, particularly in practical applications, such as driver monitoring systems. Traditional GAN-based approaches primarily focus on near-frontal LR face images, which restrict their applicability to faces with small pose variations. In fact, in the case of driver face analysis, a driver’s facial poses can vary widely due to head movements. Also, techniques like bilateral filtering are often employed to reduce ghosting and artifacts. However, these techniques can sometimes smooth out critical facial details, compromising the accuracy of facial landmark detection and the overall image quality. Therefore, in this research work, we propose a comprehensive methodology that integrates advanced computer vision and image enhancement techniques, specifically tailored for driver drowsiness detection. Furthermore, we combine the improved image with our previously developed driver state classification model to apply landmarks for real-time driver drowsiness classification.

## 3. Proposed Method

**Figure 3:**
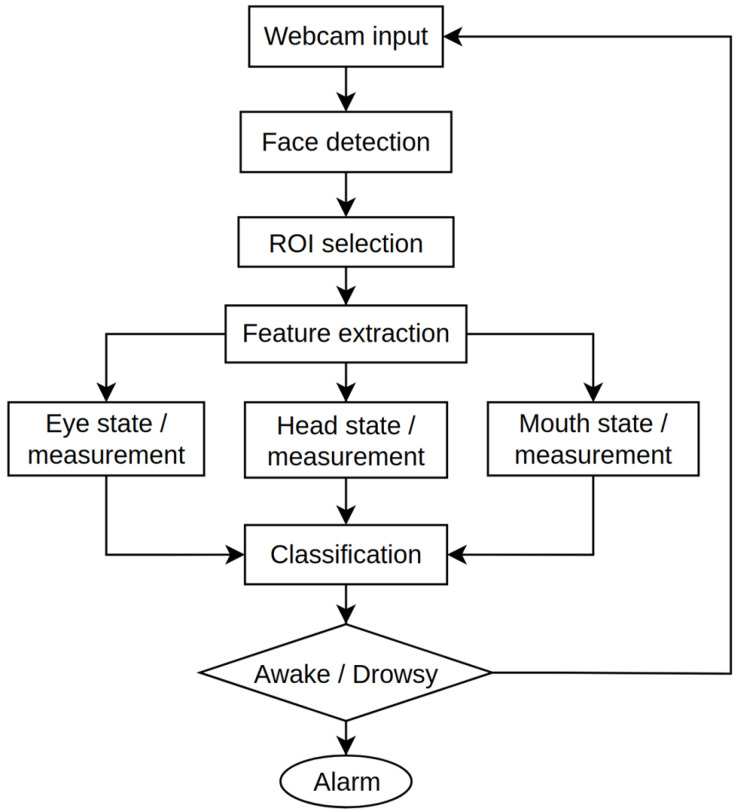
Overall process of the DDDS for behavioral measurement.

In this research work, we combined two developed models to identify driver drowsiness through face image analysis and head movement. All these approaches are included for behavioral measurements based driver drowsiness detection. Figure 3 illustrates the general process based on the behavioral-based DDDS [12].

**Figure 4:**
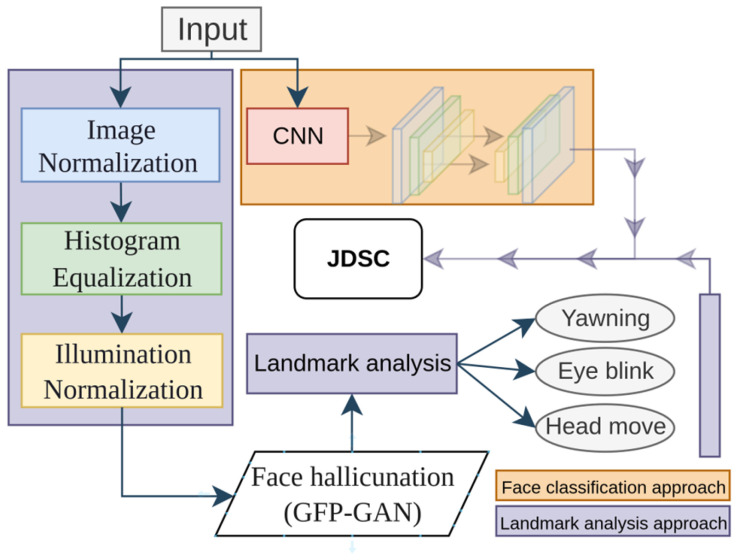
The proposed JDSC model’s combination framework to classify a driver’s face and drowsiness state.

In the first model, we developed a driver drowsiness classifier using a CNN model. Model training was performed with custom face images and open-source datasets. Model training was only performed for binary classification, with two two classes being “Drowsy” and “Awake”. Figure 4 illustrates the overall architecture of our JDSC model architecture.

This model’s development consists of two main blocks: the landmark application part and the CNN-based part. In the landmark application section, the model takes a driver’s face image as the input and processes it in three main stages: image normalization, histogram equalization, and image illumination techniques. All of these techniques serve the purpose of image enhancement. After this preprocessing step, face hallucination is applied to restore facial details, enabling better facial feature extraction and landmark application.

The JDSC model analyzes a driver’s drowsiness using joint models, such as the development of face drowsiness from 2D input image analysis and landmark-applied calculation-based analysis. The model was developed with six classes based on the head movements, yawning, and eye closure analysis separately, as shown in our previous works. Table 1 outlines a CNN architecture for driver drowsiness detection, featuring convolutional layers with increasing filters (32–128) for hierarchical feature extraction. Batch normalization stabilizes learning; max pooling reduces spatial dimensions, and dropout mitigates overfitting.

Table 2 presents the face classification model’s training dataset. The dataset includes two classes: “Awake” and “Drowsy”, with a total of 2879 images. The “Awake” class has 742 samples (582 for training, 160 for validation), while the “Drowsy” class has 2137 samples (1579 for training, 558 for validation).

The key motivation for developing this model is to optimize drowsiness detection accuracy. Though this analysis, we found that landmark application to the driver’s face is highly affected by factors such as face covering by hand or head movements from left to right. The loss of landmark application and the re-application time cause disruptions to the classification process. However, the face classification model for driver drowsiness helps reduce latency. The second section of the model, which detects the driver’s physical state from the face, focuses on applying landmarks to facial features. In this approach, we aimed to overcome the disruptions that affect or hinder facial feature detection. As mentioned above, lighting effects are a major obstacle to overcome. Therefore, we applied several image processing techniques to improve facial feature extraction and landmark application.

In addition, our method leverages a GAN-based restoration technique that incorporates generative facial priors. We enhance the resolution and quality of the input image from the webcam, facilitating more accurate and detailed facial landmark application and detection. Subsequently, the DSCM categorizes the driver’s physical state by analyzing critical indicators, such as head orientation, eye closure dynamics, and yawning.

### 3.1. Face Image Preprocessing

Image normalization equations are as follows:

First, we applied grayscale conversion:

(3) Y=0.299·R+0.587·G+0.114·B

where we convert the input color image (BGR format) to gray scale using the luminance formula. *R*, *G*, and *B* represent the red, green, and blue channels of the image, respectively. Considering *I* as the input image represented on the BGR color space, *S* is the target mean color for normalization. We compute the scaling factors $S_{R}$, $S_{G},$ and $S_{B}$ for each color channel so that their mean values match the target mean color. *μ_target_* is the target mean by the user. The scale of color channels R, G, and B changes according to their corresponding scaling factors, $S_{R}$, $S_{G},$ and $S_{B}$, respectively. Then, we obtain the normalized format for the three channels as follows:

(4) $$R=clip(R\cdot S_{R},0.255)$$

(5) $$G=clip(G\cdot S_{G},0.255)$$

(6) $$B=clip(B\cdot S_{B},0.255)$$

Thereafter, the normalized color channels were merged to obtain the final normalized image:

(7) $$I_{normilized}=merge(R_{N},G_{N},B_{N})$$

Here, the resulting normalized image undergoes illumination normalization, adjusting its brightness and color balance to match the target mean color specified by the user.

Next, we applied histogram equalization:

(8) $$g\left(z\right)=\frac{L-1}{MN}\sum_{i=1}^{z}n_{i}$$

where we compute the cumulative distribution function (CDF) of the pixel intensities in the grayscale image. We normalize the CDF to map pixel intensities uniformly across the entire dynamic image. These steps outline the process of image normalization, from converting the image to grayscale, enhancing its contrast through histogram equalization. Then, we utilized a Haar cascade face detection classifier, which provides the visual indication of the detected face based on the intensity variations in the image [55].

Through illumination normalization, we specifically focus on standardizing the lighting conditions within an image. It aims to compensate for variations in illumination intensity and color across different images or within the same image due to factors such as uneven lighting, shadows, or glare. The primary goal of illumination normalization is to improve the visibility of objects and details in images by ensuring consistent lighting conditions, which can facilitate more accurate analysis and interpretation for CV algorithms.

Given an input image I with color channels R, G  and B, the illumination normalization process can be described as follows:

We computed the mean color intensity:

(9) $$\mu_{R}=\frac{1}{N}\sum_{i=1}^{N}R_{i},$$

(10) $$\mu_{G}=\frac{1}{N}\sum_{i=1}^{N}G_{i},$$

(11) $$\mu_{B}=\frac{1}{N}\sum_{i=1}^{N}B_{i},$$

where *N* is the total number of pixels in the image.

Illumination normalization and image normalization are both preprocessing techniques used to enhance the quality and consistency of images, but they address different aspects of image variability.

### 3.2. Generative Facial Prior (GFP) GAN-Based Image Enhancing

Our research endeavors to integrate the GFP-GAN model into the process of face restoration, particularly in the context of driver monitoring systems. By leveraging the special feature transformation layers inherent in the GFP-GAN model, we aim to enhance the quality of facial images captured under adverse conditions, characterized by external factors such as traffic headlights, shadow formations, low-light environments during nighttime, and the intense illumination of sunlight. The primary objective of incorporating the GFP-GAN model in our research work is to ensure the preservation and accurate representation of facial features during the face restoration process. This is crucial for subsequent tasks such as facial feature detection, where the precise localization and recognition of facial landmarks are paramount. The GFP-GAN model’s ability to restore facial details and enhance colors with a single forward pass proves instrumental in mitigating the effects of image degradation caused by external factors, thereby facilitating more robust and reliable facial feature detection.

### 3.3. Data Collection and Model Analysis Set up

**Figure 5:**
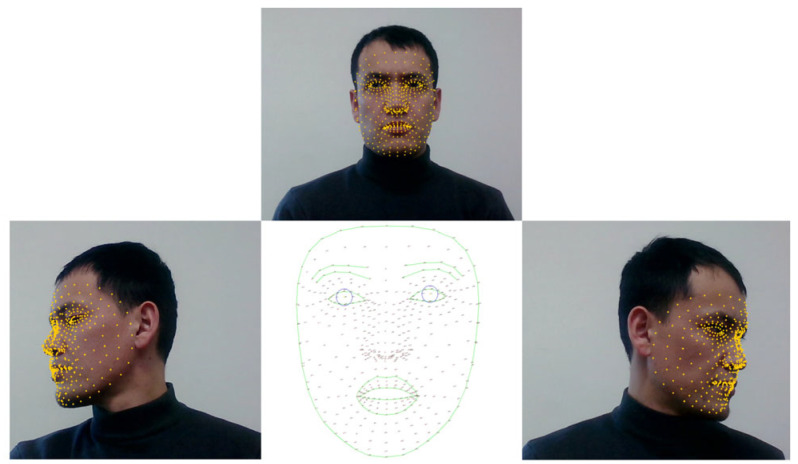
The data collection process for landmark applications based on a driver’s physical state.

For landmark-based data collection, model training and training analysis are performed using MediaPipe landmark applications. All these steps have been mentioned in our previous research work as well. The next stage is the main part of this study, where we process a back-lit, low-light, illuminated face and low-resolution face images in real-time. The previous step was developed to predict a driver’s physical state in normal conditions, where a driver’s face is not affected by outer effects. With this, we aimed to compare normal face images with distorted face images. Figure 5 depicts the procedural framework of the facial landmark application process facilitated by MediaPipe, a cutting-edge technology utilized for real-time facial analysis.

**Figure 6:**
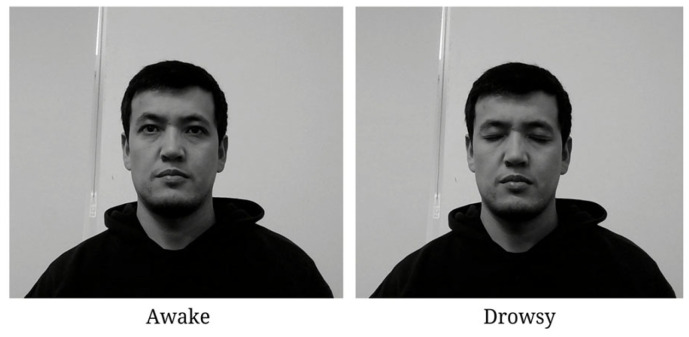
Face classification model development. Here, “0” is a label for the “Awake” class, and “1” is for the “Drowsy” class.

Through continuous data acquisition routine recording sessions, facial landmark coordinates are systematically collated onto distinct classes, as delineated previously. Notably, discernible distinctions in head movements are discerned and categorized, specifically addressing lateral deviations from the central axis. Figure 6 shows the face classification model’s development dataset in terms of two classes.

Figure 6 illustrates the measurement of the driver’s ocular state through the analysis of eye blinking. Eye blinking is counted using six landmark points related to the eyes. Therefore, accurate landmark application is considered an important factor for better measurement of driver drowsiness in scenes affected by light.

**Figure 7:**
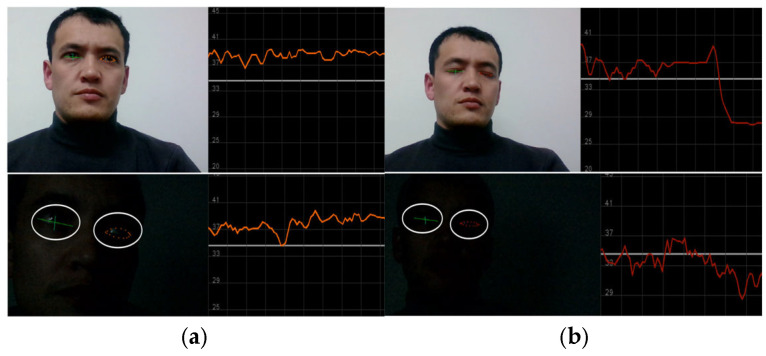
The data collection process for the eye-blinking class. (**a**) Description of the “Awake” class when an eye is open and the fluctuation line is above threshold; (**b**) Description of the “Drowsy” class when an eye is closed and the fluctuation line is below the threshold line. Below (**a**,**b**), same landmark application and class measurement are represented for nighttime scenarios.

Figure 7 also depicts the nighttime eye landmark application and blink detection. Within this graphical representation, a delineated threshold line, positioned at point 34, serves as a pivotal point between distinct ocular states, namely open and closed eye conditions. Notably, when the plotted line resides beneath the established threshold, indicating closure, the driver’s physiological condition is judiciously classified as drowsy. This discerning threshold parameter, meticulously defined within the context of ocular dynamics, plays a pivotal role in discerning and quantifying the driver’s state of alertness, thereby facilitating proactive interventions aimed at mitigating potential risks associated with drowsy driving incidents.

### 3.4. JDSC Model Evaluation

The general acceptance and agreement with factual accuracy can be evaluated through specific computational metrics. These metrics include the count of correctly identified instances within a given class, such as true positive (TP), the count of correctly identified instances outside the given class, true negative (TN), the count of instances incorrectly assigned to a class, false positive (FP), and the count of instances that were not identified as belonging to the class despite being so, false negative (FN).

**Figure 8:**
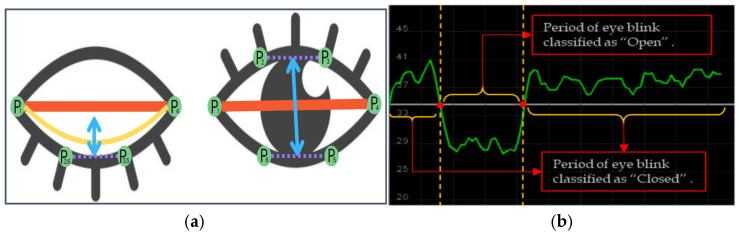
Eye landmark detection, measurement (**a**), and eye waveform fluctuation in the “open” and “close” classes. (**b**) Source image from [56].

Figure 8a shows an example of eye closure analysis. $P_{1,2,3,4,5,6}$ are landmark points of an eye.

The framework evaluation aligns with the methodologies detailed in our previously published studies [57,58].

(12) $$Precision=\frac{TP}{TP+FP}$$

(13) $$\mathrm{Recall}=\frac{\mathrm{TP}}{\mathrm{TP}+\mathrm{FN}}$$

(14) $$F1\;\mathrm{score}=\frac{2*\mathrm{Recall}*\mathrm{Precision}}{\mathrm{Recall}+\mathrm{Precision}}$$

To analyze the movement, we established a blink differentiation threshold (y = 34), as shown in Figure 7b, by determining the midpoint eye aspect ratio (EAR) scalar value. So, the EAR scalar value quantifies the degree of eye opening and closing, as detailed in Equation (6).

(15) $$EAR=\frac{\left||P_{2}-P_{6}||+||P_{3}-P_{5}|\right|}{2||P_{1}-P_{4}||}$$

Equation (15) describes the measurement of EAR, where $P_{n}$ represents the maximum landmark points. Each local position on the retina is considered with $P_{2},\;P_{3},\;P_{5},$ and $P_{6},$ which are used to calculate the vertical height of the eye, and $P_{1}$ and $P_{4}$ are used to measure the horizontal width, where all maximum landmark points are designated as positive if M(k) = 1 and as negative if M(k) = −1. The upward and downward EAR lines create a waveforms using eye blink landmark-annotated movement data ($x_{n}$ and $y_{n}$). The initial value for the threshold was set to *S*(*k*) = 1; otherwise, *S*(*k*) = 0. The threshold $T_{S}$ is estimated using the standard deviation of the first-level wavelet coefficients, as shown in the equation below:

(16) $$S\left(k\right)=\left\{\begin{array}{c} 1,\;\;if\;|\;\omega (k)\geq T_{S}|\\ 1,\;\;if\;|\;\omega \left(k\right)<T_{S}| \end{array}\right.$$

The expression *S*(*k*) represents a function that results in an output of either 1 or 0, based on whether the value of $\omega \left(k\right)$ meets or exceeds the threshold $T_{S}$. If $\omega \left(k\right)$ is greater than or equal to $T_{S}$, then *S*(*k*) = 1; otherwise, *S*(*k*) = 0. These metrics help in identifying the drowsiness and distraction levels. The framework includes a real-time alert system that notifies drivers about their physiological state, enabling timely interventions to prevent potential road hazards.

## 4. Results

In this section, we present the experimental results of our proposed methods. In the beginning, we train the face image classification model. We created a custom dataset and included a publicly available dataset related to awake and drowsy facial images. Regarding landmark-based model development, we collected facial landmark coordinates, which are regular facial features based on an analysis of a driver’s physical state in terms of three indicator factors, namely yawning, eye blinking, and head pose movements to the left and right sides, as can be seen in Figure 5 and Figure 6. All these factors play a crucial role in classifying a driver’s state as drowsy. Therefore, all classes are indicators of a driver’s physical state based on the facial feature analysis. Outside of those parameters, if the model measures facial landmark coordinates, it is classified as an awake driver.

### Experimental Dataset

Table 3 represents the experimental set up for this research. The software environment is built on Ubuntu 22.04.3 LTS, a 64-bit operating system. CUDA 12.0 is employed to leverage GPU acceleration for DL tasks, facilitating faster training and model optimization; this is especially advantageous for large language model computation. We run the system on the Linux kernel, ensuring compatibility with the latest hardware drivers and software packages.

**Figure 9:**
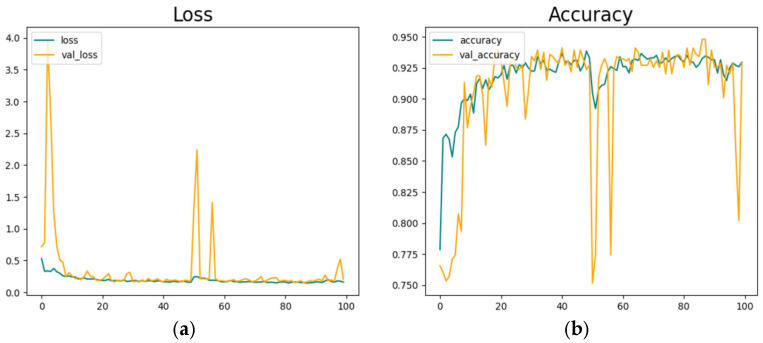
Driver face classification model development. (**a**) is depicting loss values and (**b**) is showing model’s highest accuracy results in 100 epoch training.

Figure 9 shows the classification model’s training parameters. We trained the model in 100 epochs. Figure 9a represents the model’s training loss, and Figure 9b shows the model’s training accuracy.

The model reached its best accuracy level at around 100 epochs, but after a sudden decrease and fluctuations, an accuracy of 92.5% is reached.

**Figure 10:**
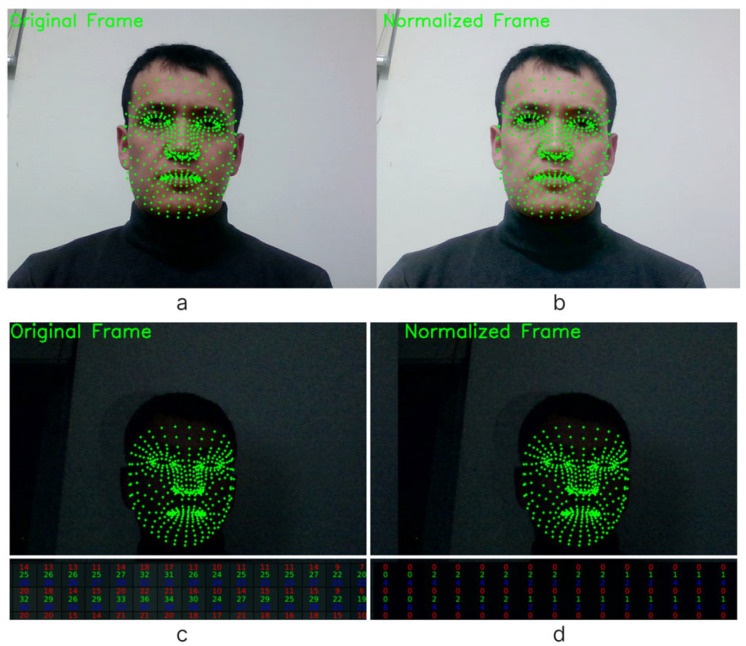
Visual representation of the original (**a**) and normalized (**b**) images with landmark applications. (**c**) An original frame without normalization, and (**d**) with a normalized facial image in the color channels.

Figure 10 presents a comprehensive visual depiction, contrasting the original and normalized facial images alongside their respective landmark applications. Figure 10a,b meticulously showcase the original and normalized facial images, respectively, with overlaid landmark annotations.

This comparative analysis underscores the efficacy of the normalization process in mitigating the adverse effects of lighting variations and enhancing facial feature discernibility. Moreover, Figure 10c,d offer a nuanced perspective of the original and normalized facial images within the context of their color channels. While Figure 10c presents the unaltered original frame, Figure 10d highlights the same frame with the applied normalization technique, highlighting improvements in color fidelity and contrast enhancement achieved through the normalization process. Through this visual representation, the salient benefits of facial image normalization, coupled with landmark applications affirming its indispensable role in refining facial analysis methodologies and strengthening the accuracy and reliability of subsequent computational analysis, are obtained.

**Figure 11:**
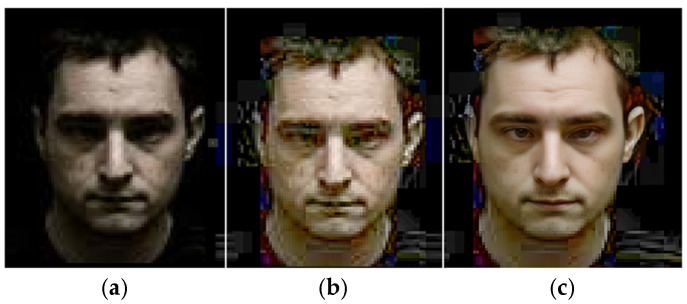
Describing a nighttime face image, (**a**) a normalized face, (**b**) and a restored face (**c**).

In Figure 11, a detailed examination is provided, delineating the descriptive characteristics of facial images captured under nocturnal conditions, subsequently normalized, and further restored with a facial restoration technique. In (a), the inherent challenges associated with low-light conditions exemplify the diminished clarity and discernibility of facial features. Next, in (b), the normalization process is applied in order to rectify luminance disparities and enhance visual clarity. This step contributes to avoiding the adverse effects of inadequate lighting effects on a driver’s face, thereby facilitating facial feature extraction and analysis. Furthermore, in (c), we can see the outcomes of a face restoration technique being applied, implemented to address the inherent limitations of low-resolution facial imagery.

Despite the inherent constraints posed by nocturnal settings, the restoration process endeavors to enhance image fidelity and restore lost details, thereby augmenting the interpretability and analytical efficacy of a driver’s face based on physiological analysis. The implementation of the face restoration technique in such scenarios signifies a pivotal advancement in computational imaging, offering pragmatic solutions to circumvent the challenges posed by suboptimal lighting conditions. Moreover, normalized and restored face images underscore the transformative impact of advanced image processing methodologies in elucidating intricate facial dynamics and facilitating nuanced analysis, particularly pertinent to eye blinking behavior analysis in nighttime face monitoring.

**Figure 12:**
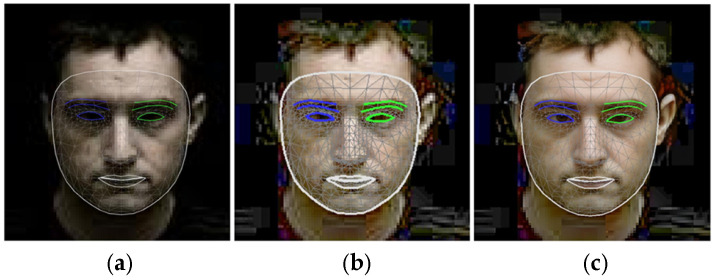
Landmark application to the nighttime view of a face (**a**), a normalized face, (**b**) and a restored face (**c**).

Figure 12 illustrates the application of landmark techniques to a nocturnal facial image, highlighting the progression from the original image (a) through normalization (b) to the restored image (c).

Notably, the restored image (c) exhibits substantial enhancements in the application of facial landmarks, particularly discernible in the refinement of eye contours, even including the iris landmark application as well.

Furthermore, comparative analysis between the normalized (b) and restored (c) representations reveals a marked improvement in edge detection along the facial perimeter, emphasizing the efficacy of the restoration process in augmenting facial feature delineation.

Table 4 presents a comparison of different driver drowsiness detection methods. Most of the studies applied a camera-based sensing method. In the architectural section, our approach combined threshold- and DL-based techniques. For threshold technique applications, we used facial landmark analysis, mostly measuring eye blinking. In DL integration, we classified a driver’s face using visual contexts, such as the “Awake” and “Drowsy” states.

Table 5 compares the performance and limitations of different methods used for driver drowsiness detection, emphasizing their respective approaches, accuracy, and constraints. T. Ahmed et al. employed a CNN model, achieving a high accuracy of 97.23%, but their approach was limited by its focus on the eye and mouth regions, potentially restricting the system’s ability to detect other critical signs of drowsiness.

R. Jabar et al. combined a CNN with Dlib facial landmark detection, achieving an accuracy of 83.33%, but their method was constrained by the reliance on frame-generated images, which could affect the quality of feature extraction and model performance. In comparison, our method offers a joint approach, utilizing both a CNN and facial landmark analysis to enhance driver drowsiness detection. The CNN model achieves 92.5% accuracy, and the facial landmark analysis-based approach achieves 97.33% accuracy. Our combined CNN plus landmark method reaches an accuracy of 94.92%, showing a 10% higher performance than the CNN plus Dlib approach. While our CNN application park is showing lower accuracy results compared to the method by T. Ahmed, our approach does not have the constraints related to focusing on specific facial features (like eye or mouth) or using generated frames.

## 5. Discussion

The detailed examination and analysis offer profound insights into the multifaceted realm of facial analysis, particularly concerning nocturnal conditions and drowsy driving detection methodologies. Looking at Figure 6, Figure 7 and Figure 9, the intricate process of facial landmark application is facilitated by state-of-the-art technologies, such as MediaPipe, as well as the meticulous measurement of ocular dynamics for drowsy driving detection. These illustrations emphasize the significance of continuous data acquisition and threshold parameters in accurately quantifying the driver’s state of alertness, thereby enabling proactive intervention to mitigate potential risks associated with drowsy driving incidents. Moving to Figure 10, the transformative impact of facial image normalization and restoration techniques in mitigating the adverse effects of low-light conditions and enhancing image fidelity is vividly demonstrated. Figure 11 provides a comprehensive visual depiction of the application of landmarks to nighttime-captured face images, highlighting the substantial enhancements needed to achieve thorough restoration processes, particularly in the refinement of facial landmarks and edge detection along the facial parameters. Conversely, camera-based approaches like those by You et al. [59] and Sharan et al. [60] leveraged DL algorithms. Their applications demonstrate higher adaptability to complex scenarios but require more computational resources, as evidenced by Sharan et al.’s deployment on a Raspberry Pi. Our method uniquely integrates both threshold- and DL-based algorithms, balancing simplicity and accuracy. In CNN model comparisons, T. Ahmed et al. [62] achieved the highest accuracy (97.23%) with a focus on specific facial regions, though it was limited by overspecialization. R. Jabbar et al.’s [63] CNN + Dlib approach achieves only 83.33% accuracy, constrained by the reliance on frame-generated images. Our approach, combining a CNN and landmark analysis, achieves robust accuracies of 92.5% and 97.33%, respectively.

## 6. Conclusions

In conclusion, the collective findings prove the indispensable role of advanced image processing techniques in advancing the frontier of facial imagery analysis, particularly in challenging nighttime settings which can be highly applicable for the analysis of drowsy driving detection using face state classification joined with facial landmark application analysis. In other words, the incorporation of facial landmark analysis demonstrates significant improvements in ocular dynamics measurement, particularly under challenging conditions like low light and nighttime scenarios after facial imagery analysis. The comparative analysis highlights the trade-offs between accuracy and computational complexity across various methods. Research findings show that the proposed approach achieves a balance between simplicity and precision, attaining accuracies of 92.5% and 97.33% in the CNN and landmark analysis, respectively.

## Figures and Tables

**Table 1:** The face classification model architecture.

Layers	Output Shapes	Parameters
conv2d (Conv2D)	(None, 256, 256, 32)	896
batch_normalization	(None, 256, 256, 32)	128
conv2d_1 (Conv2D)	(None, 256, 256, 32)	9248
batch_normalization_1	(None, 256, 256, 32)	128
max_pooling2d	(None, 128, 128, 32)	0
dropout	(None, 128, 128, 32)	0
conv2d_2 (Conv2D)	(None, 128, 128, 64)	18,496
batch_normalization_2	(None, 128, 128, 64)	256
conv2d_3 (Conv2D)	(None, 128, 128, 64)	36,928
batch_normalization_2	(None, 128, 128, 64)	256
max_pooling2d_1	(None, 128, 128, 64)	0
dropout_1	(None, 128, 128, 64)	0
conv2d_4	(None, 64, 64, 128)	73,856
batch_normalization_4	(None, 64, 64, 128)	512
conv2d_5	(None, 64, 64, 128)	147,584
batch_normalization_5	(None, 64, 64, 128)	512

**Table 2:** The face classification model’s training dataset parameters.

Dataset	Train	Val	Total
Awake	582	160	742
Drowsy	1579	558	2137
Total	2161	718	2879

**Table 3:** Software and hardware configuration.

Configuration	Versions
Hardware model Manufacture	ASRock X399 Taichi PEGATRON, Taiwan, China
Memory	32.0 GiB
Processor	AMD Ryzen ™ Threadripper ™ 1950X × 32
Graphics	NVIDIA GeForce GTX 1080 Ti
Operating system	Ubuntu 23.04
Operating system type	64-bit
Toolkit	CUDA 12.0
Kernel version	Linux 6.2.0-37-generic

**Table 4:** Proposed approach comparison with similar research works.

	Sensing Method	Algorithm Class	Embedded Algorithms
You et al. [59]	Camera-based	DL-based	No
Sharan et al. [60]	Camera-based	DL-based	Raspberry Pi
Kim et al. [61]	Camera-based	DL-based	No
Our method	Camera-based	Threshold + DL-based	No

**Table 5:** Proposed method accuracy comparison. Bold style letters representing relatively higher results.

	Approach	Accuracy	Limitations
T. Ahmed et al. [62]	CNN	97.23%	Eye/mouth focus
R. Jabbar et al. [63]	CNN + DLib	83.33%	Frame generated images
Our method	CNN	92.5%	Dataset size
	Landmark	**97.33%**	Without glass
	CNN + Landmark	**94.92%**	-

## Data Availability

The dataset is not available for sharing.
